# Ultrafine Particles: Geiser et al. Respond

**Published:** 2006-04

**Authors:** Marianne Geiser, Barbara Rothen-Rutishauser, Nadine Kapp, Peter Gehr, Samuel Schürch, Wolfgang Kreyling, Holger Schulz, Manuela Semmler, Joachim Heyder, Vinzenz Im Hof

**Affiliations:** Institute for Anatomy University of Bern, Bern, Switzerland, E-mail: geiser@ana.unibe.ch; Department of Physiology and Biophysics, Faculty of Medicine, The University of Calgary, Calgary, Canada; GSF - National Research Center for Environment and Health, Institute for Inhalation Biology, Neuherberg/Munich, Germany; Institute of Pathophysiology, University of Bern, Bern, Switzerland

Nemmar et al. were surprised that we did not cite their study in our article (Geiser et al. 2005) when we referenced state-of-the-art experiments about the translocation of ultrafine particles into secondary organs. We did not cite their human study (Nemmar et al. 2002) because in Figure 2 of their article, they presented clear evidence that a major fraction of the radio-labeled technetium-99 (Tc-99m) came off the Technegas particles. Thus, for methodologic reasons, the fraction of translocated particles could not be determined adequately and was certainly overestimated by Nemmar et al. (2002). This was recently discussed by Kreyling et al. (2004). To briefly illustrate this, we have included Figure 1. Figure 1A shows the original whole-body scintigram published by Nemmar et al. (2002) in which the salivary glands, the thyroid gland, and the urinary bladder are clearly visible, demonstrating that they contain large fractions of the Tc-99m radiolabel. This and the Tc-99m activity in the soft tissue, which shows the contour of the whole body, are clear indications of nonparticulate Tc-99m in the form of pertechnetate. Pertechnetate typically accumulates in these organs, as can be inferred from the Figure 1B, where the same pattern of radiolabel was detected after inhalation of soluble Tc-99m pertechnetate.

In the case of inhalation of nonleaching Tc-99m radiolabeled ultrafine carbon particles (Figure 1C), no activity is detectable in these organs or in the soft tissue. Figure 1C shows three images taken from the head (little larynx retention), the thorax (main carbon particle retention in lungs), and the lower abdomen, with a rather faint image of the urinary bladder. A similar pattern has been reported by Brown et al. (2002).

In addition, Nemmar et al. are interested in the surface charges of the particles we used for the *in vitro* studies, because surface charges are likely to be important determinants for the translocation of ultrafine particles as well as for their biologic effects. The polystyrene particles we used for the studies with the macrophages and erythrocytes were either uncharged, amino-modified, or carboxylate-modified (Rothen-Rutishauser B, Gehr P, Schürch S, unpublished data). The surface charges of the gold and titanium particles are not known. We found non-phagocytic uptake of ultrafine particles of all the different materials and surface charges by macrophages and erythrocytes. However, because the aim of our study was not to investigate the effects of surface charges on cellular uptake, we did not measure the actual surface charges of the particles or estimate the total number of particles within cells to quantify particle uptake. We certainly agree with Nemmar et al. on the importance of surface charges for particle-cell interaction, and we hope that we will soon find more literature published on this aspect.

## Figures and Tables

**Figure 1:**
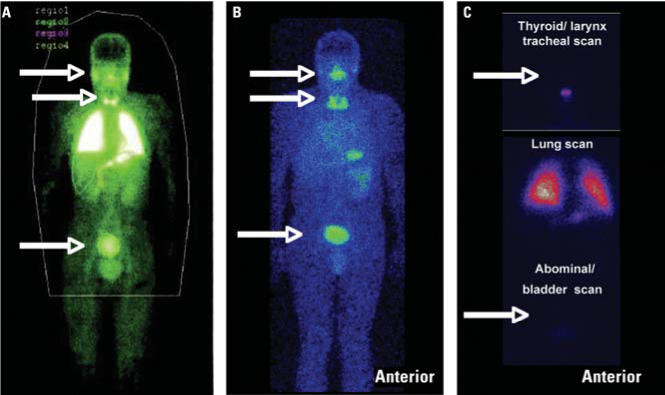
(*A*) Gamma camera image after the inhalation of Tc-99m radiolabeled ultrafine carbon particles not controlled for leaching. Reproduced from Nemmar et al. (2002) with permission from Lippincott Williams & Wilkins. (*B*) Gamma camera image after the inhalation of soluble Tc-99m pertechnetate. Reproduced from Kreyling et al. (in press) with permission from *The Journal of Aerosol Medicine*. (*C*) Gamma camera images after the inhalation of nonleaching Tc-99m radiolabeled ultrafine carbon particles.
